# The Brazilian Network for HIV-1 Genotyping External Quality Control Assurance Programme

**DOI:** 10.1186/1758-2652-14-45

**Published:** 2011-09-21

**Authors:** Denise CF Souza, Maria Cecilia A Sucupira, Rodrigo M Brindeiro, José Carlos C Fernandez, Ester C Sabino, Lilian A Inocencio, Ricardo S Diaz

**Affiliations:** 1 Brazilian STD/AIDS and Viruses Hepatitis Department, Ministry of Health, Brasilia, DF, Brazil; 2 Laboratory of Retrovirology, Federal University of Sao Paulo, Sao Paulo, SP, Brazil; 3 Laboratory of Molecular Virology, Federal University of Rio de Janeiro, Rio de Janeiro, RJ, Brazil; 4 Laboratory of AIDS and Molecular Immunology, Oswaldo Cruz Institute, Rio de Janeiro, RJ, Brazil; 5 Fundação Pro-Sangue, Hemocentro de Sao Paulo, Sao Paulo, Brazil

## Abstract

The Brazilian network for genotyping is composed of 21 laboratories that perform and analyze genotyping tests for all HIV-infected patients within the public system, performing approximately 25,000 tests per year. We assessed the interlaboratory and intralaboratory reproducibility of genotyping systems by creating and implementing a local external quality control evaluation. Plasma samples from HIV-1-infected individuals (with low and intermediate viral loads) or RNA viral constructs with specific mutations were used. This evaluation included analyses of sensitivity and specificity of the tests based on qualitative and quantitative criteria, which scored laboratory performance on a 100-point system. Five evaluations were performed from 2003 to 2008, with 64% of laboratories scoring over 80 points in 2003, 81% doing so in 2005, 56% in 2006, 91% in 2007, and 90% in 2008 (Kruskal-Wallis, p = 0.003). Increased performance was aided by retraining laboratories that had specific deficiencies. The results emphasize the importance of investing in laboratory training and interpretation of DNA sequencing results, especially in developing countries where public (or scarce) resources are used to manage the AIDS epidemic.

## Background

Since 1991, the Brazilian Government has implemented a successful policy to guarantee universal free antiretroviral therapy (ART) access to all HIV-1-infected individuals according to local guidelines. There is also a laboratory network supported by the Brazilian Ministry of Health for HIV-1 monitoring, with 90 laboratories performing CD4 and CD8+ T cell counting and 83 laboratories performing HIV-1 viral load testing for all infected individuals in the country free of charge. There is also an established network for HIV-1 genotyping, which was implemented in 2001. This network, the Brazilian Network for HIV-1 Genotyping (RENAGENO), is now composed of 21 laboratories that perform and analyze genotyping tests.

One laboratory was designated as a reference laboratory (Retrovirology Laboratory of Federal University of Sao Paulo, Brazil) that, in addition to performing genotype analyses, also performs troubleshooting, prepares quality control panels and evaluations, and performs custom analyses of samples that fail to generate a result using licensed kits. The choice of kits used by RENAGENO is made on an annual basis and is related to the price of licensed kits at the time of the decision. From 2001 to 2007, the platform used was ViroSeq^® ^v.2.6 (Applied Biosystems, CA, USA), and since 2008, the platform has been the TRUGENE^® ^HIV-1 Genotyping Assay (Siemens Healthcare Diagnostics, IL, USA). Interpretation of HIV resistance is based on a local algorithm prepared by a group of Brazilian and international experts (available at http://algoritmo.aids.gov.br/atualizacao_algoritmo/site/ and http://www.ablsa.com).

RENAGENO consists of around 300 infectious disease physicians (Reference Physicians on Genotyping Interpretation or RPGs) who are trained to act as clinical virologists and to provide expert advice on the interpretation of HIV resistance tests and antiretroviral salvage therapy. The work flow of RENAGENO is as follows: (i) the attending physician orders a genotyping test and completes a form containing clinical data, including current and past antiretroviral exposure and lab results, from the patients; (ii) the request goes to the local RPG, who determines if the request fulfills the Brazilian guidelines for the use of a genotyping test; (iii) if authorized by the RPG, a sample is collected and sent to a local RENAGENO lab; and (iv) the result is sent to the RPG, who provides a written recommendation for salvage therapy to the attending physician according to the his/her interpretation of the case.

RPGs convene once a year for four days to be retrained as a part of a continuous education programme led by experts in the field appointed by individuals from the STD/AIDS and Viral Hepatitis Diseases Department of the Brazilian Ministry of Health. New RPGs are trained in a separate four-day meeting before entering the programme. RPGs are volunteers who are an important part of RENAGENO in terms of authorizing the request and the use of resistance tests *vis-a-vis *the impact of expert advice on the performance of salvage therapy (HAVANA) [1] and the influence that HIV-1 resistance tests have in the decision-making process of the physician [2].

RENAGENO had performed 54,594 tests as of December 2009 and is now set to perform 25,500 genotyping tests/year. We have implemented a quality control and assurance system to monitor the performance of the 21 laboratories that comprise the RENAGENO network. We report here on the methodology used, as well as the performance of the laboratories, in the first five external quality control evaluations (EQAs) performed in 2003, 2005, 2006, 2007 and 2008.

## Methods

Five external evaluations of the quality panels (EQA 1-5) were employed from 2003 to 2008. In each year, between 10 and 21 laboratories were evaluated (14 laboratories were evaluated in 2003, 15 in 2005, 16 in 2006, 21 in 2007, and 10 in 2008). The overall criteria for evaluating the participating laboratories are provided in Table 1. Briefly, it was considered important to evaluate: (i) the ability of purifying RNA and PCR amplify the samples; (ii) the absence of PCR carry over; (iii) the ability to obtain sequences of all involved fragments; (iv) the ability to obtain good quality sequences; (v) and the ability to identify resistance-related mutations in involved codons.

**Table 1:** Criteria for laboratory evaluation

		Sample 1	Sample 2	
**Qualitative variables**				

Positive PCR amplification				

		15	15	30

Genetic diversity of sequenced samples				

Less than 2.2%		10	10	20

**Quantitative variables**				

Number of obtained sequences				

4		2	2	

5		4	4	

6		8	8	

7		10	10	20

Correct interpretation of mutations (codons)				

Protease 1		1	1	

2		1	1	

3		1	1	

4		1	1	

5		1	1	10

Reverse transcriptase 1		1	1	

2		1	1	

3		1	1	

4		1	1	

5		1	1	10

Quality of sequences (Phred quality scores)				

100% above 30		5	5	

80-99% above 30		2.5	2.5	10

**Total**				**100**

(The viral load of sample 1 is 2000 copies/mL and the viral load of sample 2 is 10,000 copies/mL)

The weights provided for each category were arbitrarily chosen according to the level of importance the members of the panel that prepared the interpretation gave to each step of the test. One sample with low viral load and one sample with intermediate viral load was included in each evaluating panel. The same weights was given to both low and intermediate viral load samples since it was considered to be equally important for well-trained laboratories to obtain good quality results in any type of samples. Correct interpretation of resistance *vis-a-vis *available antiretrovirals were not evaluated since interpretations may change over time and there are many available algorithms for resistance interpretation.

## Samples used

EQA 1, 3, 4 and 5 used panels made from clinical samples presenting specific drug resistance mutations and polymorphisms in the reverse transcriptase and protease regions of *pol*. For EQA_2, infectious clones with specific antiretroviral mutations were constructed. Samples were diluted with seronegative plasma and adjusted to have viral loads of approximately 2000 (sample 1) and 10,000 (sample 2) copies of HIV-1 RNA/mL of plasma. Sample 1 and sample 2 came from different donors and had different mutation profiles, and mutation profiles differed among different EQA panels. All samples were from clade B HIV strains. No samples without HIV have been used. Informed consent was obtained from patients that provided the samples. The determination of the "true profile" of the clinical samples was performed by at least two bidirectional independent sequencing reactions.

## Performance evaluation

### Qualitative criteria

#### Sensitivity

The objective was to evaluate the ability of a laboratory to amplify and sequence two panels of samples; one panel has samples that contain 2000 copies HIV RNA/mL and the other panel has samples that contain 10,000 copies RNA/mL. The smaller viral load is representative of the lower detection limit of the commercial kits used.

#### Specificity

The objective was to evaluate the ability to amplify and generate sequences similar to the known profile of a specific strain. Variation was accepted between sequences if it was within the expected genetic diversity of the quasispecies of the specific viruses or the expected misincorporation rate of the PCR polymerase (~1/500 misincorporations) [3]. All generated sequences from different evaluated laboratories were aligned using ClustalX software, and a distance matrix was generated using DNADIST software. We considered the maximum genetic diversity of the analyzed region to be 2%, and therefore, for a 1.3-kb fragment, a maximum difference of 29 nucleotides would be expected (26 mutations based on normal HIV-1 genetic diversity and three mutations based on the polymerase misincorporation rate). Thus, if the generated sequenced presented a distance that was 0.022 greater than the consensus sequence (2.2% distance), the possibility of PCR carryover was considered.

### Quantitative criteria

#### Number of generated sequences

The result was usually obtained by generating five to seven sequences that cover the *pol *region of interest with the necessary redundancy, including some sequences from the 5' to 3' direction and vice versa, based on the assumption that a small number of generated sequences (less than five) correlates with a greater possibility of misinterpretations.

#### Quality of generated sequences

The quality of generated chromatograms was interpreted according to the fluorescence generated by each terminal nucleotide. This fluorescence value was measured by the Phred quality score (The Phred-Phrap Package software). The chromatograms of good quality were those with intensity signals over 30, which corresponds to 99.9% accuracy of the base call (1/1000 probability that the base is called incorrectly). For this analysis, the percentage of nucleotides of each of the seven fragments with an emission signal over 30 was considered. Each laboratory was awarded 5 points if 100% of nucleotides scored above 30, 2.5 points if 80-99.9% of nucleotides scored above 30, and zero points for percentages below 80%.

#### Analysis of mutations in the reverse transcriptase and protease regions

In this analysis, each participating laboratory was evaluated on its ability to identify amino acid mutations at five key positions in each genomic region from sequences generated at the reference laboratory (Table 1).

### Statistical analysis

To evaluate the general performance of labs over time, the Kruskal-Wallis test was used for analysis of mean values.

## Results

Table 2 describes the percentage of laboratories that achieved the correct outcome on each of the criterion as a percentage of the maximum score. The scores of genetic diversity and all quantitative variables refer only to samples that did not fail to amplify (Table 2). Laboratories not able to amplify any of the two samples received the score of zero (Table 3). A positive PCR result was obtained 92.9% of the time when higher viral load samples were analyzed and 90.5% (Table 2) of the time when lower viral loads were used, and this performance was stable over time (data not shown). According to the expected genetic diversities of the viruses, PCR carryover has not been detected in any sequenced samples. Drug resistant mutations present in each panel containing viruses and sequences sent for analysis is depicted in Table 4.

**Table 2:** Overall performance of laboratories according to each evaluated criterion as a percentage of the maximum score

		Sample 1	Sample 2	
**Qualitative variables**				

Positive PCR amplification				

		90.5%	92.9%	91.7%

Genetic diversity of sequenced samples				

Less than 2.2%		100%	100%	100%

**Quantitative variables**				

Number of obtained sequences				

4		0%	0%	

5		6.0%	4.8%	

6		6.0%	3.6%	

7		75.9%	80.7%	88.6%

Correct interpretation of mutations (codons)				

Protease	1	0%	0%	

2		1.2%	1.2%	

3		0%	0%	

4		1.2%	1.2%	

5		85.7%	88.1%	89.3%

Reverse transcriptase	1	0%	0%	

2		1.2%	1.2%	

3		0%	0%	

4		1.2%	1.2%	

5		85.7%	88.1%	89.3%

Quality of sequences (Phred quality scores)				

100% above 30		22.7%	25.0%	23.8%

The results correspond to the mean values of the five evaluations. The viral load of sample 1 is 2000 copies/mL and the viral load of sample 2 is 10,000 copies/mL

**Table 3:** Overall performance evaluation in number of points (out of a maximum of 100 points) and standard-deviation (SD) for each evaluation

LAB	EQA1	EQA 2	EQA 3	EQA 4	EQA 5
1	30	0	NA	NA	NA

2	96	80	80	88.2	90

3	100	96.5	75	96.8	86.1

4	96	99.2	95	98.2	88.7

5	50	84.3	95	98.2	84.8

6	0	47.1	49	83.2	23.4

7	90	99.6	97.5	97.9	88.4

8	90	100	NA	NA	NA

9	30	NA	88	97.5	88.7

10	0	98.2	95	97.5	86.7

11	100	100	100	75.9	90

12	95	95	95	98.2	88.7

13	95	NA	85	98.2	88.7

14	NA	92.3	95	98.2	NA

15	100	95	95	97.9	NA

16	NA	100	45	98.2	90

17	NA	NA	93	98.2	86.1

18	NA	NA	NA	97.9	85.4

19	NA	NA	80	97.9	89.7

20	NA	NA	NA	10.5	88.7

21	NA	NA	NA	98.2	88.7

22	NA	NA	NA	97.9	90

23	NA	NA	NA	97.5	74.4

Mean (SD) 69.4 (± 38.73) 84.8 (± 36.60) 85.1(± 16.77) 91.5 (± 19.47) 84.1 (± 15.11)

% > 80 64.3 85.7 81.1 90.5 89.5

LAB = the code number for each evaluated laboratory. EQA = External Quality Control Evaluation. EQA1 was performed in 2003, EQA2 in 2005, EQA3 in 2006, EQA4, in 2007, and EQA5 in 2008. NA = not available due to the absence of participation of a specific laboratory in the network or in the evaluation. % > 80 refers to the percentage of participating labs that scored more than 80 points. Laboratories that were not able to PCR amplify both samples received a score equal zero.

**Table 4:** Drug resistance mutations present in each EQA panel

	VL1		VL2	
	
	PROTEASE	REVERSE TRANSCRIPTASE	PROTEASE	REVERSE TRANSCRIPTASE
EQA1	WT	WT	L10V, M36M/I	WT

EQA2	V77I	M184V, T215Y, K103N, A98G, P225H	I54V, L63P, A71V, V82A, L90M	WT

EQA3	L10F, K20R, L33F, I54L, V82A	Y181C, R211K, T215C	L10V, L63P, V77I	M41L, M184V, Y188L, L210W, T215Y

EQA4	L10F, L63P, V77I, I84V, I93L	D67N, K70R, M184V, T215T/I, K219E	K20T, M46I, L63P, A71T, N88G	M41L, K103N, V118I, M184V, T215Y

EQA5	E35D, I62V, V71T, I84I, L90L/M	K103N, M184M, R221K, L214F, T215L	E35D, I62V, A71T, I84I, L90L/M	K103N, M184M, R211K, L214F, T215L

(VL1 = viral load 1 of 2000 copies/mL and VL2 = 10,000 copies/mL. WT = wild type virus. A total of five codons at the protease and five codons at reverse transcriptase were selected for analysis. In the absence of drug resistance mutations, randomly selected wild type codons were chosen for the correct interpretation of mutations analysis. In case of ambiguities - mixed viruses population, i.e, M36M/I - we scored as correct only labs that reported the presence of both amino acids.)

The overall performance scores of laboratories in the EQA are depicted in Table 3. The data indicate that the overall scores improved over time. As shown in the table, 64.3% of laboratories scored over 80 points in 2003, 81.7% did so in 2005, 81.1% in 2006, 90.5% in 2007, and 89.5% in 2008 (Kruskal-Wallis statistic on medians among groups over time = 16.16, p = 0.003). No clear reason has been detected to justify some laboratories' score fluctuation between panels as seen for laboratories 3, 6, 11 and 16.

Retraining has been performed in laboratories 5, 6 and 9 after the first evaluation, in laboratory 16 after the third evaluation and in laboratory 20 after the fourth evaluation according to the specific needs of each laboratory detected in the EQA evaluation (data on file). Retraining included principles of preventing PCR carryover, specific laboratory technical procedures and sequence editing processes. A small group of technicians from the reference laboratory and technicians from the company that produced the licensed genotype kits were responsible for conducting the retraining.

## Discussion

We describe here a methodology that was created to evaluate Brazilian laboratories that assist the Brazilian Ministry of Health in its genotyping network. All participating laboratories are public laboratories, most of which are located at public universities and have primary activities related to research as opposed to clinical care assistance. Using this novel method to evaluate laboratories, we introduced some variables to refine the evaluation of the laboratories and laboratory personnel with respect to the ability of each laboratory to generate results and to evaluate the sensitivity of each laboratory system using low viral load samples as described in the Methods section.

With this method, we were also able to evaluate the quality of the generated sequences by measuring the intensity of the signal, and we were able to evaluate the possibility of generating incorrect results by analyzing the genetic diversity of generated sequences. It has been pointed out that one of the weak points in homogeneity between labs may be the proficiency in the sequence editing process among different individuals [4]. Furthermore, we encouraged lab personnel to interpret the sequences and evaluated personnel in their ability to recognize expected mutations, including insertions and deletions, among sequences generated elsewhere (data on file).

This external quality control evaluation also enables us to detect, in an active manner, laboratories that need retraining in a more dedicated fashion or to exclude specific under-performing labs from the network. Specialists from the diagnostic companies that sold the kits performed retraining, upon request from the Ministry of Health HIV/AIDS Department, when scores were found to be low. As noted in Table 3, low performance was not common, and in general, labs showed improvement after specific retraining.

One interesting study evaluating the proficiency of the sequencing editing process between different laboratories produced panels of HIV-1 isolates from subtypes B, C and F [4]. These strains presented specific drug resistance mutations and viral loads between 5000 and 10,000 copies/mL [4]. We chose to use clinical samples rather than viral clones for four of the five evaluations in this study in the interest of maintaining a realistic scenario and increasing the complexity of laboratory manipulation and interpretation as suggested by others [5,6]. Nonetheless, our second evaluation used RNA viral constructions and the performances of the laboratories were, in general, similar to those observed in subsequent evaluations (EQA 3, 4 and 5). The results show that, overall, the average performance of the laboratories was adequate, with 82.4% of evaluations resulting in scores exceeding 80 points.

Although we did not detect PCR carryover in these evaluations, we consider that this issue deserves careful attention and intervention. To further improve the ability to detect PCR carryover or sample mislabelling on a daily basis, a software tool was created to evaluate similarities between generated sequences in real time http://bioinf.aids.gov.br/. With this tool, every generated sequence was compared with the sequences previously generated in a specific laboratory, and close similarities would raise a flag and precipitate investigation and corrective measures, including retraining.

As noted in Table 2, the reported quality of sequences according to the Phred quality scores was not high. For the purpose of this evaluation, we intentionally set the bar too high to encourage laboratories to pursue improvements in the quality of sequences, as well as to increase the number of redundant sequences available in the system, as described in the Methods section.

## Conclusions

In our network, we use licensed kits to generate our results in our certified laboratories. As mentioned previously, the ViroSeq^® ^v.2.6 was used from 2001 to 2007, and the kit currently used is the TRUGENE^® ^HIV-1 Genotyping Assay. No changes in the performance of laboratories were detected during or after this transition, according to our evaluation. Another large international genotyping proficiency programme also demonstrated that different technologies using ViroSeq, TruGene or in-house assays successfully genotyped panel samples, although variations in the quality of results have been observed between laboratories [7].

Furthermore, we believe that our results emphasize the importance of investing in employee training and in increasing interpretation skills, especially in developing countries that are using public (or scarce) resources to manage the AIDS epidemic.

## Competing interests

DS and LI are former employees of the Brazilian STD/AIDS and Viruses Hepatitis Department, Ministry of Health, Brasilia, DF, Brazil. MS, JF and RD are consults of Brazilian STD/AIDS and Viruses Hepatitis Department, Ministry of Health, Brasilia, DF, Brazil. RB and ES have no competing interests.

## Authors' contributions

DS: Design of the study, data analysis. MS: Molecular virology laboratory assistance, data analysis. RB: Virology laboratory assistance. JF: Molecular virology laboratory assistance. ES: Design of the study. LI: Design of the study. RD: Design of the study, Manuscript preparation. All authors have read and approved the final manuscript.
